# 

**Published:** 1911-11-18

**Authors:** 


### CONTENTS.

| | PAGE |
|---|---|
| The Insurance Bill and the Hospitals ... ... | 165 |
| Hospital Managers and Probationers' Contracts | 166 |
| Problems of Modern Psychiatry—II. ... ... | 173 |
| Medical Legal Points ... ... ... ... ... ... | 175 |
| The Making of a Modern Hospital—V. ... | 177 |
| Hospital Architecture and Construction ... | 179 |
| The New Boarding-Out Order ... ... ... | 180 |
| The Defective and Dependent—II. ... ... | 181 |
| Legal Notes for Institutional Workers ... ... | 182 |
| Reports on the Hospitals of the United Kingdom ... ... ... ... ... ... | 183 |
| By-ways of Medical Relief ... ... ... | 184 |
| Maternity and Mothering ... ... ... ... | 185 |
| Institutional Housekeeping ... ... ... ... | 186 |
| Our Bureau of Information ... .. ... | 187 |
| The British Hospitals Association in Glasgow | 188 |
| Hospital and Institutional News ... ... ... ... | 167 |
| The Scottish Hospitals and the Bill—The Italian Red Cross at Tripoli—London's New Medical Officer—Dr. Newsholme on Vital Statistics and Officers of Health—The New Secretary of St. Paul's Hospital—Hospitals and School Children—The New Out-Patients' Department at Paddington Green—The Control of London's Casual Wards—West Norfolk and King's Lynn Hospital—The Presidency of the College of Surgeons—Dental Inspection and Anæsthetics—Hospital Abuse in India—Christmas for the London Cripples—Gifts to Hospitals by Aerial Post—The Medical Council Election—The Chronicler of Guy's—The Standard of Appeal Writing—King George and King Edward's Fund—The Latest Effort at Hartshill—An Extraordinary Protest Meeting—A Warning to Liverpool Chaplains—The Box System—A Use for Criticism—Maggoty Patent Food—The Duties of a Hospital Pharmacist—This Week's Drug Market | |
| The Editor's Letter Box ... ... ... ... ... | 188 |
| Florence Nightingale Memorial ... ... ... | 190 |
| Appointments ... ... ... ... ... ... ... | 190 |
| The Week's Novelties ... ... ... ... ... | 190 |

Supplement—National Insurance and the Voluntary Hospitals.

